# A Bibliometric Study on Global Snakebite Research Indexed in Web of Science

**DOI:** 10.3389/ijph.2023.1606311

**Published:** 2023-10-30

**Authors:** Chuanzhu Lv, Zihui Lei, Yanlan Hu, Xinyue Song, Juntao Wang, Wenjie Hao, Lanfen He, Yu Chen, Xiaotong Han, Yong Gan, Shijiao Yan

**Affiliations:** ^1^ Emergency Medicine Center, Sichuan Provincial People’s Hospital, University of Electronic Science and Technology of China, Sichuan, China; ^2^ Research Unit of Island Emergency Medicine, Chinese Academy of Medical Sciences (No. 2019RU013), Hainan Medical University, Haikou, China; ^3^ Key Laboratory of Emergency and Trauma of Ministry of Education, Hainan Medical University, Haikou, China; ^4^ Department of Social Medicine and Health Service Management, School of Public Health, Huazhong University of Science and Technology, Wuhan, China; ^5^ International School of Public Health and One Health, Hainan Medical University, Haikou, China; ^6^ Department of Emergency, Hainan Clinical Research Center for Acute and Critical Diseases, The Second Affiliated Hospital of Hainan Medical University, Haikou, China; ^7^ Department of Emergency Medicine, Hunan Provincial Key Laboratory of Emergency and Critical Care Metabolomics, Hunan Provincial Institute of Emergency Medicine, Hunan Provincial People’s Hospital/The First Affiliated Hospital, Hunan Normal University, Changsha, Hunan, China

**Keywords:** snakebite, bibliometrics, visualization map, snakebite envenoming, antivenom

## Abstract

**Objective:** To conduct a bibliometric analysis of the global snakebite literature to provide a reference for the future development of snakebite research.

**Methods:** The Web of Science citation analysis tools, VOSviewer and CiteSpace V were used to carry out the bibliometric analysis of the literature and generate visualization maps.

**Results:** The number of publications has increased at a considerably accelerated rate in the past 8 years. Nine distinct cooperation clusters were formed between institutions and countries. Keyword clustering yielded nine well-structured clusters covering two major topics, i.e., snakebite envenoming and antivenom. Burstiness detection revealed eight keywords with strong emergence, including neglected tropical diseases, Elapidae, Viperidae, and Russell’s viper, which have sustained popularity up to the present.

**Conclusion:** Current research on snakebites has gradually garnered attention from the academic community. Cooperation papers between nations severely affected by snakebite and those with higher economic status received more attention. The continued exploration of therapeutic mechanisms, the development of antivenoms or alternative medicines, and primary prevention of snakebites to ensure the safety of populations in impoverished regions should be prioritized by international scholars. The epidemiological evidence and the timely translation of research findings should be valued by policymakers.

## Introduction

Snakebite refers to the disease caused by the venom of a poisonous snake after it bites a human, resulting in acute systemic poisoning. Poisoning is primarily caused by three types of toxins: neurotoxins, hemotoxins, and mixed toxins [1]. Snakebites have a sudden onset and a wide range of clinical manifestations and can lead to disability or even be life-threatening for individuals if not treated promptly or if treated improperly. There are approximately 5.4 million snakebite incidents worldwide each year, resulting in a substantial socioeconomic impact. However, tropical developing countries, which are most severely affected by snakebites, lack robust recording methods, and many victims do not have access to hospitals, leading to a severe underestimation of the occurrence of snakebites [2]. In 2017, snakebite envenoming was classified by the World Health Organization (WHO) as a neglected tropical disease requiring critical action [3].

Anti-snake venom serum (antivenom) is the only effective cure. Due to its high price and lack of preparation, most hospitals have insufficient reserves, and thus, patients are unable to receive appropriate and timely treatment. In many countries, due to political, cultural, supply, or economic reasons, individuals with snakebites fail to access high-quality antivenom and instead seek alternative therapies, wasting precious time and increasing the likelihood of adverse outcomes [4]. Snakebites often occur among impoverished farmers in agriculture and animal husbandry sectors, and poor medical conditions and severe sequelae have substantial impacts on impoverished communities, perpetuating the cycle of poverty for victims. The 2030 Agenda for Sustainable Development proposes to eradicate all forms of poverty worldwide, making snakebite research crucial for achieving the goals of the agenda. The aim of this study was to use a bibliometric approach to carry out a visual analysis of the global snakebite literature to explore the key topics of snakebite research as well as the dynamic and static structure of its history; thus, insights into future research directions can be provided [5].

## Methods

### Data Sources

The Web of Science Core Collection database was selected to perform a search using “snakebite” as the author keyword on 24th April 2023. The types of studies were limited to research articles and review articles, with no restriction on publication year. A total of 905 relevant articles were retrieved. After excluding duplicate and incomplete articles, 900 articles published between 1997 and 2023 were included in this study. The articles, including “full records and cited references” were annotated and exported in plain text format.

### Research Methods

The analysis retrieval and citation reporting functions provided by Web of Science were used to conduct a statistical analysis of the number of publications and citation frequencies. VOSviewer (1.6.18) was used to build a cooperation network map of publishing institutions and countries. CiteSpace V (5.7. R5) was used for visual analyses of keyword co-occurrence and literature clustering. The specific parameters for constructing visual maps were as follows: time span, 1997–2023; time increment, 1 year; cosine algorithm for link strength; node selection criterion, g-index ≥ 25; and Pathfinder as the pruning method.

### Statistical Methods

In the cooperation network map constructed using VOSviewer, the size of the node represents the number of publications, the nodes of different colours represent different cooperation clusters, and the distance between the nodes and the thickness of the connecting lines represent the level of cooperation between institutions. In the keyword co-occurrence map constructed using CiteSpace, the size of the node represents the number of publications, the betweenness centrality measures the importance of the knowledge element of the node, and a node with high betweenness centrality is highly cited and serves as pivotal content connecting two different fields. Concurrently, keyword burstiness detection reflects the frontier of research in a certain period, where nodes characterized by high burstiness garner widespread attention in that timeframe. The modularity value (Q value) and the average silhouette value (S value) in the upper left corner of the map are used to evaluate the clustering effect. A Q value > 0.3 indicates a significant clustering structure, and an S value > 0.7 indicates high clustering efficiency and sufficiently convincing clustering results [6].

## Results

### Analysis of Publication Year and Journals

The earliest recorded publication on snakebite in the Web of Science Core Collection database dates to 1997, with a total of 900 relevant articles between 1997 and 2023. Excluding the articles published in 2023, the number of publications showed a slow upward trend from 1997 to 2014. Starting in 2015, there was a substantial increase in the publication rate, with articles from the subsequent 8 years (587 articles) accounting for 65.22% of all publications. The trend of publications from 1997 to 2023 is displayed in Supplementary Figure S1.

The citation status of snakebite studies was analysed using the citation analysis function of Web of Science. There was a total of 8,599 citations for the snakebite studies, with an average of 12.84 citations per article and an H-index of 47. The 900 articles included in this study were published in 303 different journals. Among them, TOXICON was the journal that had the highest number of publications, with 148 articles and an average of 15.75 citations per article. Among the top 10 journals in terms of the number of publications, TOXICON ranked second in the average number of citations per article. JOURNAL OF ETHNOPHARMACOLOGY had the highest average number of citations per article, i.e., 31.21, with a main focus on traditional herbal medicines for snakebite treatment [7] (Supplementary Figure S2).

### Analysis of Institutions and Countries

#### Analysis of the Number of Publications and Cooperation Networks of Institutions

The institutions with the highest numbers of publications on snakebite are mostly located in tropical regions where venomous snakes are more prevalent. Among them, the University of Costa Rica ranks first in the number of publications, with an average of 38.05 citations per article, which ranks first among the top five institutions in terms of the number of publications, indicating that the impact of its publications on snakebite is the greatest. The Liverpool School of Tropical Medicine ranks second in terms of both the number of publications and average number of citations per article (Supplementary Table S1).

**FIGURE 1:**
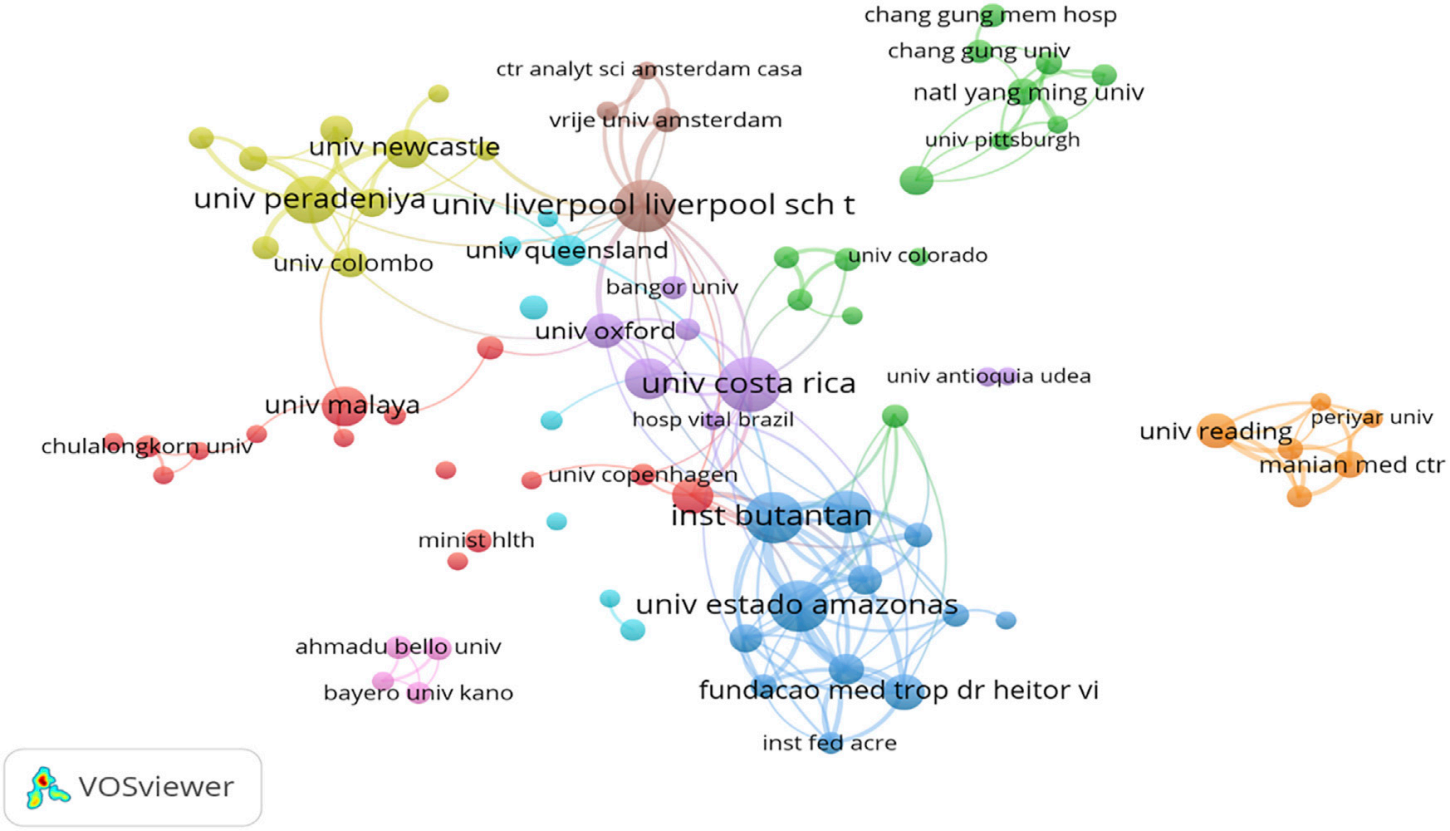
Institutional cooperation network map (China, 2023).

A cooperation network between institutions is constructed in Figure 1 by clustering the co-authoring institutions. There are a total of nine cooperation clusters. Among them, four cooperation clusters formed with the University of Costa Rica, the Liverpool School of Tropical Medicine, Instituto Butantan at Universidad del Estado del Amazonas, and the University of Queensland as cores are closely connected to each other. Three other cooperation clusters formed with the University of Peradeniya, National Yang Ming University in Taiwan, and the University of Malaya as cores have relatively few connections with the other clusters. The cooperation cluster with Bayero University and University of Reading as the core is relatively independent.

#### Analysis of the Number of Publications and Cooperation Networks of Countries

The United States (US) has carried out the most research on snakebites, but it ranks only fourth in terms of the impact of research literature among the top five countries with the highest number of publications. Australia and the United Kingdom (UK) have higher impacts regarding their research literature, ranking first and second, respectively. Specifically, both the United Kingdom and Australia have four articles each with more than 100 citations, focusing on anti-coagulopathy caused by snakebite and the synergistic prevention of snakebite [8, 9]; uneven resource allocation and clinical effects, and adverse reactions to antivenoms [4, 10]; and therapeutic drugs for snake venom [11] (Supplementary Table S2).

**FIGURE 2:**
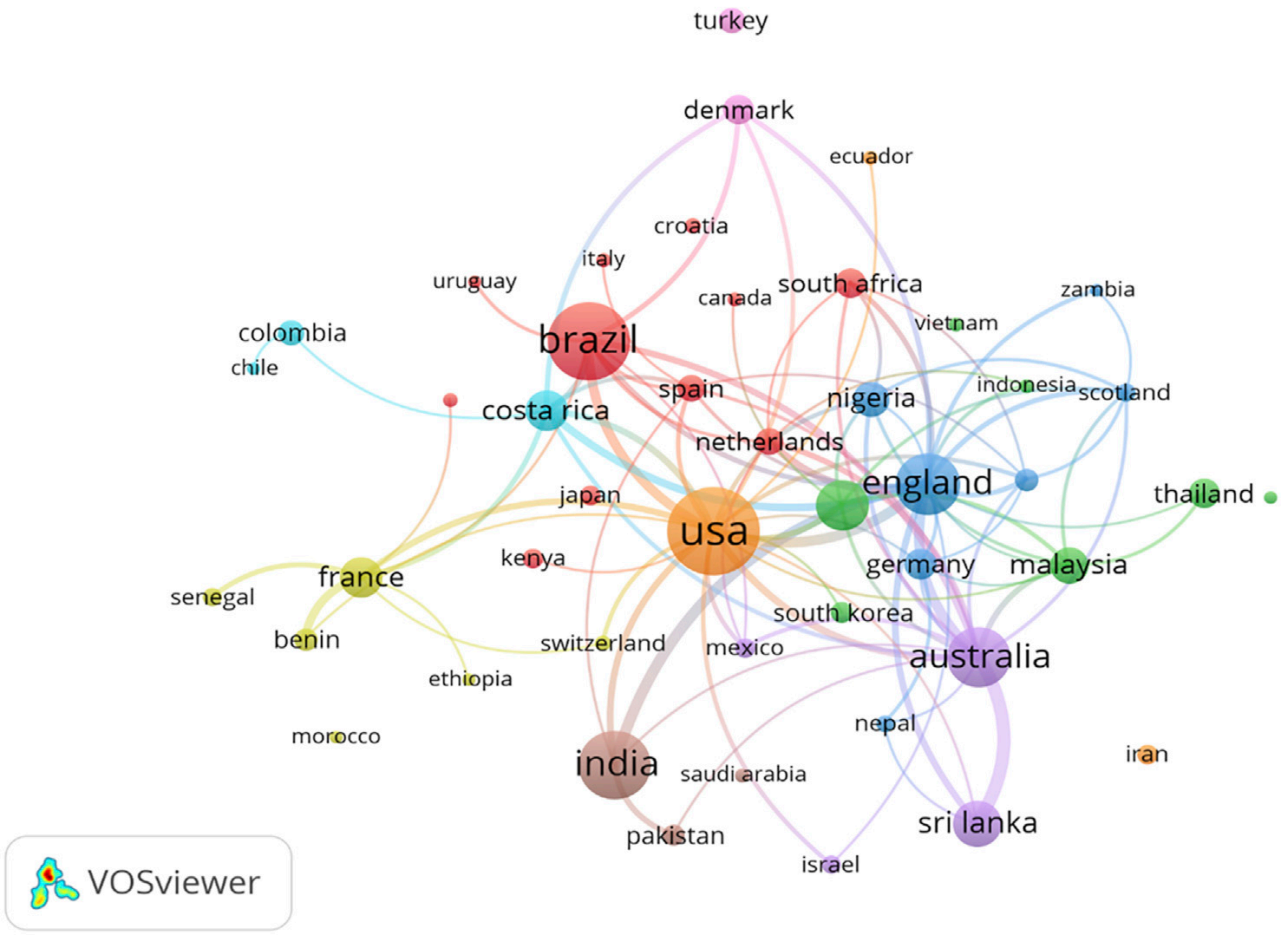
Cooperation network map for countries (China, 2023).

Figure 2 illustrates the cooperation networks among countries by co-authorship cluster. A total of nine cooperation clusters have formed among countries. The United States, Brazil, United Kingdom, India, and France have each formed cooperation clusters with single countries as cores; other cooperation clusters include two or more cores, such as the clusters between Australia and Sri Lanka, between Denmark and Turkey, between Costa Rica and Colombia, and between Malaysia, China, and Thailand. There are close connections among all nine cooperation clusters of countries.

### Keyword Analysis

#### Keyword Co-Occurrence Analysis

Using CiteSpace to extract and analyse the keywords from titles, abstracts, and author keywords as shown in Table 1, the top 10 high-frequency keywords were snakebite, envenomation, antivenom, venom, epidemiology, coagulopathy, snake, Bothrops, acute kidney injury, and Africa. Keywords with betweenness centrality higher than 0.1 are generally considered to be important and represent significant topics in previous studies. There were six keywords in the snakebite literature with betweenness centrality higher than 0.1, i.e., snakebite, envenomation, antivenom, venom, epidemiology, and coagulopathy. A review of the relevant literature with high frequency and high betweenness centrality keywords revealed that the research mainly focuses on clinical progress in, prevention strategies for and epidemiological trends of snakebite; challenges in obtaining antivenom; treatment effects; and alternatives approaches.

**TABLE 1:** Ranking of keywords with high frequency and high betweenness centrality (China, 2023).

Rank	High-frequency keywords	Frequency	Keywords with high betweenness centrality	Centrality
1	Snakebite	751	Snakebite	0.81
2	Envenomation	290	Envenomation	0.50
3	Antivenom	267	Antivenom	0.47
4	Venom	112	Venom	0.32
5	Epidemiology	58	Epidemiology	0.14
6	Coagulopathy	43	Coagulopathy	0.12
7	Snake	42	Snake	0.10
8	Bothrops	31	Medicinal plant	0.08
9	Acute kidney injury	30	Africa	0.07
10	Africa	28	Sri Lanka	0.06

#### Keyword Cluster Analysis

**FIGURE 3:**
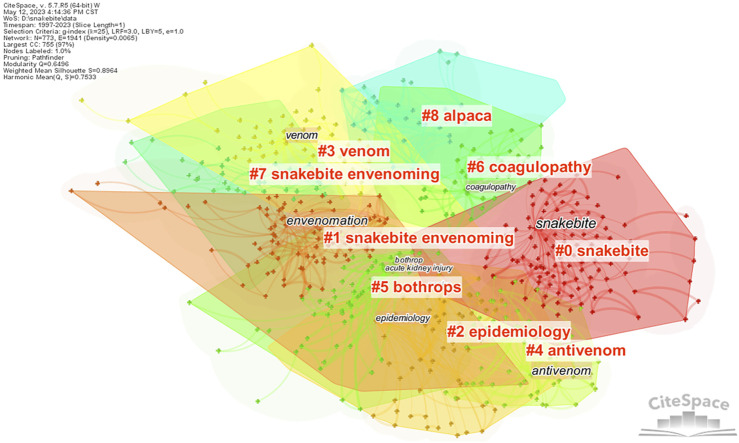
Cluster map of keywords (China, 2023).

**FIGURE 4:**
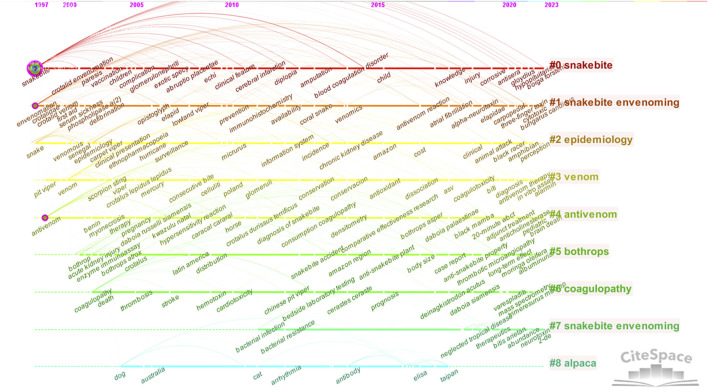
Cluster timeline of keywords (China, 2023).

A clustering network of keywords was constructed using CiteSpace to better reflect the important topics of previous research in the field of snakebite. As shown in Figure 3, the previous literature forms a total of nine well-structured clusters. The nine topics are arranged in order according to the size of the clusters, focusing on snakebite, snakebite envenoming, epidemiology, venom, antivenom, Bothrops, coagulopathy, snakebite envenoming and alpacas. By analysing the timeline of clustered keywords in Figure 4, we can further clarify the main content and differences in the eight topics. Although both Clusters 1 and 7 have snakebite envenoming as the topic, Cluster 1 focuses on the toxicology of snake venom, and Cluster 7 focuses on the complications of acute poisoning. Cluster 0, the largest cluster, focuses on the clinical manifestations and treatment of snakebites. In addition, Cluster 2 focuses on the prevalence of different snake species, Cluster 3 focuses on the diagnosis of snakebite envenoming, Cluster 4 focuses on the preparation and clinical application of antivenom, Cluster 5 focuses on the distribution, venom, and clinical aspects of Bothrops snakes, Cluster 6 focuses on the clinical manifestations caused by coagulopathy, and Cluster 8 focuses on the preparation of antivenom from alpacas. In addition, the timeline analysis revealed that, except Cluster 8, the remaining clusters still garnering greater attention. Currently, research is increasingly focusing on the toxicology and therapeutic mechanisms of snakebites, with a gradual refinement of attention to snake venom diversity.

#### Keyword Emergence Analysis

**FIGURE 5:**
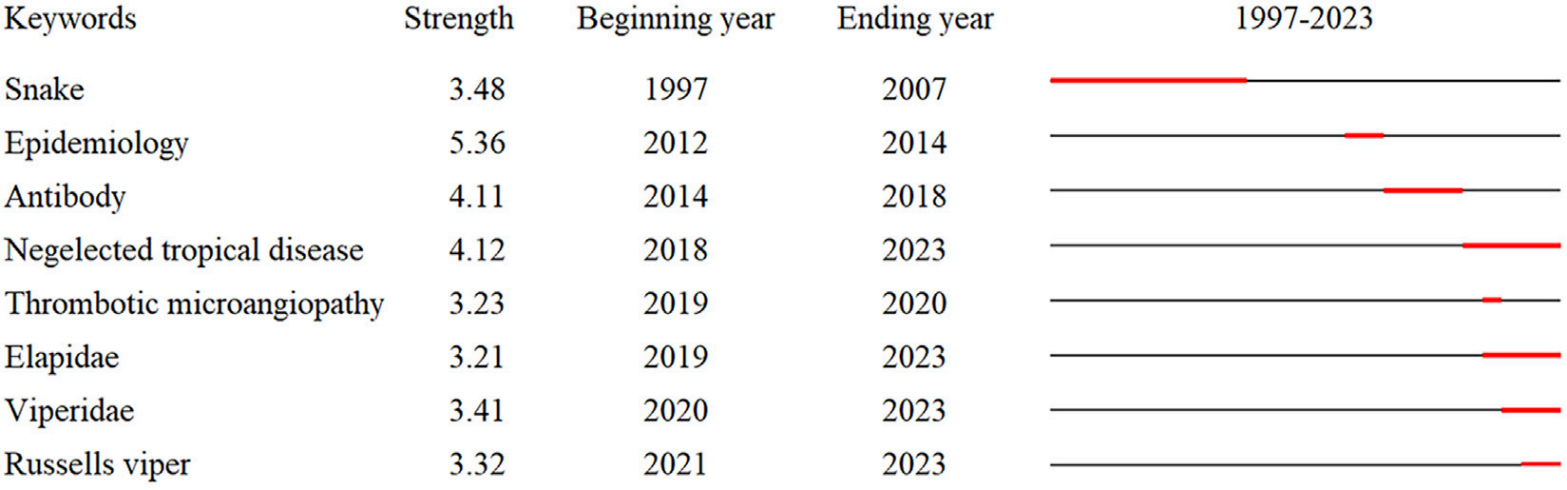
Burstiness detection map of keywords (China, 2023).

CiteSpace was used to perform burstiness detection on extracted keywords to further clarify the core of research in different periods and to understand the current research frontiers. A total of eight keywords were identified (Figure 5); in Figure 5, the red line segments indicate the years when the number of publications with specific keywords surged. In terms of emergence intensity, epidemiology, neglected tropical disease, and antibody have a high intensity, and in terms of emergence time, the current hotspots mainly include neglected tropical disease, Elapidae, Viperidae, and Russell’s viper. Snakebite envenoming was included in the WHO’s list of neglected tropical diseases (Category A) in 2017, which is consistent with the timing of its emergence as a keyword. Elapidae and Viperidae include most of the modern venomous snake species, and Russell’s viper has drawn attention as a highly aggressive venomous snake in Asia that overlaps with human habitats.

## Discussion

Since the WHO listed snakebite envenoming as a Category A neglected tropical disease in 2017, there has been a substantial increase in publications. This study, based on a bibliometric analysis of 900 snakebite-related papers, provides preliminary insights into publication trends, cooperation networks, and research themes. Subsequently, we will further discuss these findings in the context of the included literature.

### Snakebite Research Has Garnered Gradually Increasing Attention, Leading to the Formation of a Large-Scale Cooperation Network Among Institutions and Countries

Exploration of the snakebite literature in the Web of Science Core Collection revealed a consistent and rapid increase in the number of publications since 2015, indicating a growing interest among researchers in the field of snakebite. These articles have an average of 12.84 citations per article and an H-index of 47. The impact of the literature on snakebite, among the neglected tropical diseases listed by the WHO, is relatively low; therefore, there is room for further expansion and in-depth exploration of snakebite research. In terms of journals, TOXICON is in Journal Citation Reports (JCR) quartile 3 for toxicology and pharmacology, but it has a high number of publications and a high literature impact in the field of snakebite. The JOURNAL OF ETHNOPHARMACOLOGY, which has a higher literature impact, is in JCR quartile 1 for integrative and complementary medicine and plant sciences, aligning with its focus on traditional herbal medicines in the snakebite literature.

An analysis of the cooperation networks of institutions and countries revealed that most of the institutions and countries with the greatest attention to snakebites are located in tropical regions. Furthermore, the Liverpool School of Tropical Medicine in the UK has a high number of publications and a considerable impact in the field of snakebite, particularly with highly cited articles (literature reviews and randomized clinical trials) in high-quality journals [4, 12]. The cooperation network of countries indicates that the countries in the centre of the map are more closely connected with other countries. These countries are severely affected by snake envenoming or have higher levels of economic development and larger populations, such as the United States, the United Kingdom, China, Australia, Brazil, India, and Costa Rica. Although international cooperation has resulted in the creation of highly cited clinical literature, it is essential to acknowledge that regions most severely affected by snakebites are predominantly remote areas across developing countries, where healthcare resources and reporting capabilities are lacking. Consequently, the global landscape of snakebite remains a formidable challenge, underscoring the continued necessity for sustained international investment and academic exchange in snakebite research.

### Snakebite Research Has Focused on Two Major Topics, Snakebite Envenoming and Antivenom, and Future Hotspots Include the Diagnosis, Treatment, Prevention and Control of Snakebite

According to the co-occurrence analysis and cluster analysis of literature keywords using CiteSpace, seven topics related to snakebite envenoming and two topics related to antivenom appeared in nine clusters, but antisera, antivenoms, and broad-spectrum antivenoms appeared in seven different clusters. Therefore, the relevant literature in the field of snakebites has focused on two major topics, namely, snakebite envenoming and antivenom. The following topics have been the focus of previous studies in the field of snakebite envenoming: clinical progress [13], clinical diagnosis [8], epidemiological trends [14], toxicological mechanisms [15], and prevention and response strategies [9]. In the field of antivenoms, the main focus of previous studies has been on practical barriers to access [4], preparation methods [16], clinical effects [10, 12], and alternative treatments [17].

The timeline diagram of keyword clustering revealed that recent studies have paid more attention to the exploration of other snake species, the toxicological testing of snake venoms, clinical long-term evaluations, the preparation of broad-spectrum antivenoms, and paediatrics; the trends for these keywords serve as a reference for future snakebite research. A secondary search of keywords with strong emergence further revealed that the diagnosis, treatment and prevention and control of snakebite are the current hot topics of interest, for example, studies of the evidence-based diagnosis and treatment of snakebite [18, 19], enzyme inhibitors [20, 21], snake venom proteomics [15], recombinant venom serum antibodies [16], broad-spectrum antivenom [22], clinical effects of antivenom [23], and macroeconomic and social factors [24]. Research on snakebite diagnosis, treatment and prevention and control is in line with the WHO’s overall 2021–2030 approach to neglected tropical diseases. Given the inadequacy of efficient and widely applicable treatment protocols in remote regions, research efforts aimed at safeguarding the health rights of these populations need to be enhanced.

### Current Snakebite Guidelines Lack Epidemiological Evidence, and Enhanced Policy Translation of Research Findings Should Be Emphasized

In the review of published snakebite guidelines in China [25, 26], we found that these guidelines predominantly focus on the diagnosis and treatment of snakebites but less on prevention and control strategies. Furthermore, there is a scarcity of evidence regarding population epidemiology in these guidelines. The WHO’s strategy for snake envenoming prevention and control indicates that the average cost of snakebites was US$ 124 per treatment, which is much higher than that of other neglected tropical diseases [27]. In severely affected countries, the inability to afford high-quality antivenoms has led to health adverse health effects among impoverished populations. Therefore, snakebite prevention should take precedence, research on the prevalence of snakebites and health education strategies needs to be valued by policymakers, and health administrative departments need to establish standardized documents based on high-risk groups’ characteristics to promote snakebite prevention and control at the grassroots level.

In accordance with the increasing research interest in snake envenomation, investigations into envenomation mechanisms, treatment protocols, and antivenom production have been progressively advancing and increasing. The treatment methods for snakebites are continually expanding, and the quality of care is consistently improving. Timely clinical translation of research findings is imperative in this context. In the future, it is important to enhance international collaboration aimed at reducing the cost of snakebite treatments, thereby ensuring timely and sufficient treatment for impoverished populations.

### Innovations and Limitations of This Study

This is the first study to carry out a bibliometric investigation of the snakebite-related literature worldwide. By constructing relationship maps for knowledge elements, the overall situation of publications on global snakebite research was clarified, the popular and key topics in the literature were summarized, and possible directions for future research and policy on snakebite were identified. However, there are still some limitations to this study. Specifically, the literature analysed in this study was obtained by searching authors’ keywords and then reviewed to ensure consistency of the research topic of snakebites. However, not all relevant literature was searched, and hence, the results of this study may deviate from reality. We merged synonyms and near-synonyms during the analysis, but there may still have been incomplete merging. As a result, there may have been numerical differences in the betweenness centrality analysis and burstiness detection of keyword nodes.

### Conclusion

In this study, we conducted a bibliometric analysis of the snakebite literature in the Web of Science Core Collection. We identified a current trend of increasing interest in the field of snakebite, a relatively low impact of the literature, cooperation networks of countries and institutions, and important topics and frontier trends in previous research. We revealed a correlation of the impact of the snakebite literature with international cooperation, and the continued investment and academic exchange in countries facing severe impacts hold great significance. Early-stage studies in this field have focused more on the toxicological mechanisms, clinical manifestations, epidemiological trends, prevention and control strategies, barriers to access, preparation methods, clinical efficacy, and alternatives to antivenom. Early-stage studies addressed some specific mechanisms of snakebite envenoming. Future focus areas will shift towards the treatment and prevention of snakebite envenomation. Ensuring the safety of populations in impoverished regions is paramount; thus, the continued exploration of therapeutic mechanisms and the development of antivenoms or alternative medicines should be prioritized by international scholars. In addition, the application of epidemiological evidence in preventing snakebites, and the translation of research findings on low-cost snakebite treatment should be valued by policymakers.
