# Supplementary material for: A Bibliometric Study on Global Snakebite Research Indexed in Web of Science
Source: Int J Public Health. 2023 Oct 30;68:1606311. doi: 10.3389/ijph.2023.1606311 (PMC10641039; doi:10.3389/ijph.2023.1606311)
Supplement: Supplementary file 1 [file DataSheet1.docx]

Figure S1. Distribution of publications on snakebite by year from 1997 to 2023

Figure S2. Ranking of journals by number of publications on snakebite

Table S1. Ranking of top institutions by the number of publications

| Rank | | Institution | Number of publications | Number of citations per article | Country |
| --- | --- | --- | --- | --- | --- |
| 1 | University of Costa Rica | | 40 | 38.05 | Costa Rica |
| 2 | Liverpool School of Tropical Medicine | | 37 | 26.18 | United Kingdom |
| 3 | Instituto Butantan | | 36 | 17.20 | Brazil |
| 4 | Universidad del Estado del Amazonas | | 35 | 8.23 | Brazil |
| 5 | University of Peradeniya | | 31 | 8.94 | Sri Lanka |

Table S2. Ranking of countries with high numbers of publications

| Rank | Country | Number of publications | Number of citations per article |
| --- | --- | --- | --- |
| 1 | United States | 176 | 13.01 |
| 2 | Brazil | 136 | 13.92 |
| 3 | India | 112 | 9.26 |
| 4 | United Kingdom | 86 | 22.51 |
| 5 | Australia | 85 | 23.89 |
